# Genetic Variants in Cytokines *IL-10* and *IL-17A* Are Associated with the Risk and Severity of Sporadic Parkinson’s Disease in Mexican Patients

**DOI:** 10.3390/life15091480

**Published:** 2025-09-20

**Authors:** Nancy Monroy-Jaramillo, Alberto Ortega-Vázquez, Marisol López-López, Laura Virginia Adalid-Peralta

**Affiliations:** 1 Departamento de Genética, Instituto Nacional de Neurología y Neurocirugía Manuel Velasco Suárez, Mexico City 14269, Mexico; 2 Departamento de Sistemas Biológicos, Universidad Autónoma Metropolitana Unidad Xochimilco, Mexico City 04960, Mexico; aortega@correo.xoc.uam.mx (A.O.-V.); mlopez@correo.xoc.uam.mx (M.L.-L.); 3 Laboratorio de Reprogramación Celular, Instituto de Fisiología Celular UNAM en el Instituto Nacional de Neurología y Neurocirugía, Mexico City 14269, Mexico; laura.adalid@innn.edu.mx

**Keywords:** Parkinson’s disease, IL-10, IL-13, IL-17A, inflammation

## Abstract

Background/Objective: Parkinson’s disease (PD) is the most common neurodegenerative movement disorder, in which the inflammatory hypothesis posits that dysregulation of the immune system is an important factor in its etiology. Based on our previous findings, we aimed to explore if promoter functional genetic variants in cytokines encoded by *IL-10* and *IL-17A*, and a coding variant in the *IL-13* gene, confer risk to the inflammatory profile of PD patients. Methods: For population genetic purposes, DNA samples from 239 patients with sporadic PD and 84 healthy controls were included and genotyped for *IL-10_*rs1800896, *IL-10_*rs1800872, *IL-13*_rs20541, *IL-17A*_rs2275913, and *IL-17_*rs8193036 variants by allelic discrimination in real-time PCR. All participants were Mexican Mestizos (MMs). Gene frequencies and haplotype association analyses were assessed. Then, a subgroup of 26 untreated PD patients and 21 controls were selected from this cohort for detailed biochemical and clinical data and evaluated by multivariate correlation analysis at the baseline and one year post-dopaminergic replacement treatment. Results and Conclusions: *IL-17A*_rs8193036 was found to be associated with increased PD risk (*p* < 0.010, OR = 1.91, CI_95%_ = 1.22–2.98). The genetic variants *IL-10*_rs1800896, *IL-10*_rs1800872, and *IL_17A*_rs8193036 were related to the inflammatory process in sporadic MM PD patients, suggesting that they may play potential roles in the development and severity of the disease.

## 1. Introduction

Parkinson’s disease (PD) is the second most prevalent neurodegenerative disorder; the majority of cases are sporadic and multifactorial, and its etiology has not been clearly established [1,2]. However, evidence shows that the inflammatory process and immunological dysfunction (including dysregulated cytokine secretion) play a relevant role in the pathogenesis of sporadic PD [3,4,5,6]. Indeed, neuroinflammation exacerbates dopaminergic neurodegeneration with an inflammatory cascade involving microglial activation and the marked secretion of pro-inflammatory cytokines. The alterations of inflammatory biomarkers in the blood of patients with PD also indicate the peripheral involvement of PD pathogenesis [6]. Inflammatory cytokines (e.g., TNF, IL-6, IL-1β, and others) are increased in both the brain and peripheral immune system of PD patients, correlating with disease severity and disability [3,4].

In PD, dysregulated cytokine secretion is driven by a profound imbalance between pro-inflammatory and regulatory immune cells, contributing to disease progression [7,8]. It has been described that PD patients display an expansion of Th1 and Th17 cells, both in treatment-naïve individuals and those receiving dopaminergic therapy, accompanied by a reduction in Treg populations [9]. The cellular ratio highlights this imbalance, as a markedly elevated Th17/Treg ratio underscores the predominance of an inflammatory over regulatory response in PD. Functionally, this profile is associated with the excessive production of pro-inflammatory cytokines, such as IFN-γ (Th1), IL-17A (Th17), and TNF-α, along with a reduction in anti-inflammatory cytokines, including IL-4 (Th2) and IL-10 [9]. The net result is a systemic and central inflammatory milieu that actively contributes to dopaminergic neurodegeneration [7,8,9].

Some reports have observed increased serum IL-10 levels in patients with PD compared with control participants [6,10], while other studies have not indicated any changes in serum and cerebrospinal fluid IL-10 levels in patients with PD [11,12]. IL-10 is one of the immune molecules that have been proposed as potential peripheral biomarkers in PD [6]. IL-10 acts mostly as an anti-inflammatory cytokine by its regulatory effects on several pro-inflammatory cytokines [13,14,15,16,17,18,19]. Therefore, lower levels of the *IL-10*_rs1800896 variant can have a protective effect on susceptibility to PD [13,14,15,16,17,18,19]. IL-10 also possesses properties to induce cytokine production, such as IFN-γ by natural killer cells, and promotes B-cell survival, proliferation, and differentiation [20]. Approximately 75% of the level of IL-10 production is genetically determined [20]. IL-10 gene expression is largely dependent on the single-nucleotide variants (SNVs) c.-1082 (rs1800896), c.-819 (rs1800871), and c.-592 (rs1800872), located in the promoter region [13,14,15,16,17,18,19].

The action of IL-13 as an anti-inflammatory cytokine is through a heterodimeric receptor complex which is expressed in dopaminergic neurons and includes its subunit, IL-13Rα1. This subunit lies within the *PARK12* locus of PD susceptibility [21] and contributes to the loss of dopaminergic neurons during inflammation, suggesting that IL-13 may be involved in the etiology and/or the progression of PD [22]. The Gln144Arg substitution of the *IL-13*_rs20541 variant lies in the critical region for interactions between IL-13 and its receptors and may have an impact on IL-13-mediated signaling [21,22].

Sommer et al. (2019) found that Th17 cells accelerated the death of midbrain neurons induced by PD patient-derived induced pluripotent stem cells, indicating that IL-17A may be involved in PD pathogenesis [7]. Th17 cells have been shown to infiltrate into the brain parenchyma through the disrupted blood–brain barrier (BBB) in PD [21,23]; IL-17A is considered the hallmark cytokine of Th17 cells [21]. Evidence in rodent models suggests that IL-17A accelerates neurodegeneration in PD depending on microglial activation and at least partly TNF-α release [24,25]. In addition, dopaminergic neurodegeneration, motor impairment, and BBB disruption were relieved in the IL-17a knockout mice [24]. Nevertheless, decreased plasma IL-17A levels have been reported in PD patients [26,27]. In this study, we included the *IL-17A*_rs2275913 and *IL-17A*_rs8193036 variants, which are in the promoter region and could regulate gene expression [28].

IL-10 can directly suppress the production of IL-17A, while some IL-17-producing Tregs can also co-secrete IL-10, suggesting a complex, context-dependent, and sometimes reciprocal regulatory relationship [29]. Interactions between IL-13 and IL-17A have also been described, with IL-17A generally enhancing IL-13’s pathological actions, while IL-13 can suppress IL-17A’s beneficial effects [30].

It has been proposed that certain genetic differences in the immune response, especially in the production of anti-inflammatory and pro-inflammatory cytokines, could influence the risk of developing this disease [3,4]. Although there is evidence of this link, results have been inconsistent. In addition, research studies tend to include PD patients under treatment, which does not allow for determining whether there are pre-diagnostic levels of inflammatory biomarkers that contribute to the risk or clinical outcomes in PD patients [5]. Preceding work of the group reported altered IL-13 and IL-17A levels in untreated Mexican Mestizo (MM) PD patients, suggesting a deficient suppression of the pro-inflammatory response [26]. Then, after 2 years of dopaminergic replacement treatment, these patients showed increased Tc17 cell levels and an imbalanced immune response [31]. These alterations favor an enhanced inflammatory response and PD progression. Based on these findings, we wondered whether this regulatory impairment could be partly explained by the presence of genetic variants in the cytokines IL-10, IL-13, and IL-17A in PD patients.

Despite all this evidence, the causal relationship between circulating pro- and anti-inflammatory cytokines and the risk and clinical variables of PD has not been yet elucidated, and there is only one single study on Europeans [32]. Moreover, although a few genetic variants have been associated with inflammation and PD, the results cannot directly apply to the MM population. The *IL-10_*rs1800896, *IL-10_*rs1800872, *IL-13*_rs20541, *IL-17A*_rs2275913, and *IL-17_*rs8193036 variants selected in this study have been associated with the serum/plasma levels of the corresponding cytokine (as detailed in Supplementary Table S1) and have been previously related to various diseases of an inflammatory nature in the MM population [33,34,35,36,37,38]. With the above stated, we aimed to determine the association of these five functional variants in the *IL-10*, *IL-13*, and *IL-17A* genes with the inflammatory profiles of and clinical measures in MM patients with sporadic PD.

## 2. Materials and Methods

For the present study, we included DNA samples from a biobank of the Department of Genetics (Instituto Nacional de Neurología y Neurocirugía Manuel Velasco Suárez, INNN), as follows: for population genetics purposes, 239 consecutive and unrelated patients with a clinical diagnosis of MM sporadic PD patients and 84 MM healthy controls were included.

Then, a subgroup of participants was selected from this cohort for the detailed biochemical and clinical analyses. This subgroup was called the “clinical group” and consisted of 26 untreated PD patients who were seen at the outpatient clinic of the INNN, and 21 paired by sex and age, apparently healthy controls at enrollment. From all individuals blood plasmas were available for analytical measurements (see below). Patients were diagnosed by movement disorder specialists according to the Movement Disorder Society clinical diagnostic criteria for PD [39].

The remaining 213 patients and 63 controls were only used for the population genetics part of the study, giving a total sample study of 239 PD patients and 84 controls. All participants (controls and patients, including those from the biobank) reported both parents and four grandparents of MM origin to ensure ethnic homogeneity. The present study was carried out in accordance with the latest version of the Declaration of Helsinki, and the study design was reviewed and approved by the local ethical and research committees. Written informed consent was obtained from all participants after the nature of the procedures had been fully explained (approval codes INNN_38/19 and 95/14; biobank samples from INNN_110/09 and 42/10).

### 2.1. Molecular Analysis

Genomic DNA from peripheral blood mononuclear cells (PBMCs) of 239 PD patients and 84 controls was isolated using standard procedures. Two SNVs of the *IL-10* (rs1800896, rs1800872), one of the *IL-13* (rs20541), and two of the *IL-17A* (rs2275913 and rs8193036) gene were genotyped by TaqMan^®^ probes on a QuantStudio-5™ real-time PCR system (Thermofisher, Waltham, MA, USA). Characteristics of the genetic variants are presented in Supplementary Table S1.

After performing population genetics analysis (see below) in all samples, different genetic models were applied for each genetic variant: codominant, dominant, recessive, overdominant, and additive models. Moreover, to determine whether certain haplotypes may be associated with PD in an MM population, linkage disequilibrium (LD) and haplotype blocks were calculated using Haploview [40]. The pairwise LD analysis was assessed by D′ and r^2^ measures.

### 2.2. Biochemical Analysis

The following biochemical and clinical data were uniquely available for the 26 untreated PD patients and 21 controls (clinical group). Cytokine levels were determined via an enzyme-linked immunosorbent assay (ELISA) in plasma samples of the clinical group to explore the overall effect of immune response at the baseline and at one-year of dopaminergic replacement treatment (DRT) in PD patients. A hemogram was included for the total blood cell count, and the clinical data of PD patients was evaluated by movement disorder specialists, who used the Hoen and Yahr scale (HY), the Movement Disorder Society-sponsored version of the Unified Parkinson’s Disease Rating Scale (MDS-UPDRS) total scale, the Schwab and England Activities of Daily Living (SE-ADL) scale, and Beck’s Depression Inventory (BDI). The neutrophil–lymphocyte index (NLI) and the systemic immune-inflammation index (SII) were calculated using the counts of neutrophils, lymphocytes, and platelets from a routine blood test, as reported [41]. The additional data of these samples have previously been published [26,41].

### 2.3. Statistical Analysis

Allelic and genotypic frequencies were calculated using Haploview [40]. Hardy–Weinberg (H–W) equilibrium and differences in the frequencies of the variants among groups were determined using the χ^2^ test. Pairwise linkage disequilibrium (LD) was estimated using the Lewontin D′ measure (D0) between the genetic variants of *IL-10* and haplotype analysis with Haploview version 4.1 (Broad Institute of Massachusetts Institute of Technology and Harvard University, Cambridge, MA, USA).

Descriptive and inferential statistics were used to describe the demographic characteristics of the study groups. Associations in the clinical group (26 PD patients and 21 controls) were assessed using the student’s *t*-test and ANOVA for continuous variables, while chi-square (χ^2^) and Fisher’s exact tests were used for categorical data.

The multivariate regression analysis included inflammation indices, with biochemical and clinical parameters. Covariates included in the analyses were age and sex for all participants, while plasma cytokine levels (at the baseline in untreated patients and one year post-DRT) were only considered for PD patients. All statistical tests were performed using SPSS^®^ Statistics version 22.0 software (IBM, New York, NY, USA) and R version 3.5.1 (R Core team, Vienna, Austria) [42]. A *p*-value of <0.05 was considered statistically significant.

## 3. Results

### 3.1. Clinical and Biochemical Parameters in Patients and Controls

Demographic, biochemical, and clinical characteristics of the participants are shown in Table 1. PD patients and controls were comparable by sex and age in the overall sample and in the clinical group. In addition, the participants for the analysis of biochemical and clinical variables showed similar weight, height, and body mass index (BMI). Plasma levels of IL-17A were found to be increased in controls vs. PD patients (*p* < 0.022); on the contrary, IL-13 levels showed a trend toward being higher in PD patients compared to controls (*p* = 0.2). Added to this, controls exhibited higher cholesterol levels than PD patients (*p* = 0.048), and female patients showed lower uric acid levels (*p* < 0.001) and higher cholesterol levels (*p* = 0.01) vs. male patients.

### 3.2. Distribution of Genetic Variants in the Participants

Allele and genotype frequency distribution for the five genetic variants was calculated for PD patients and controls, and frequencies were then compared between (i) total PD patients and total controls, (ii) treated patients and untreated patients, and (iii) untreated PD patients in the clinical group and controls in the clinical group (Table 2). The distribution of the studied genetic variants in *IL-10*, *IL-13*, and *IL-17A*_rs2275913 was similar in PD patients and healthy controls (n = 239 vs. n = 84) (Table 2), except for the *f*(T) of the *IL-17A*_rs8193036 variant, which showed a significant difference between groups regarding the CC genotypes and allelic frequencies (*p* < 0.010), with an OR = 1.91 (CI_95%_ = 1.22–2.98) and an RR = 1.16 (CI_95%_ = 1.06–1.27), *p* = 0.004. The subsequent genotype comparisons between TT and CC + CT yielded an OR = 1.69 (CI_95%_ = 0.99–2.87), RR = 1.14 (CI_95%_ = 1.00–1.29), and that between CC and CT + TT yielded an OR = 4.32 (CI_95%_ = 1.29–14.50), RR = 1.28 (CI_95%_ = 1.13–1.44). Homozygous genotype CC was more frequent in patients, while the genotype TT was more frequently observed in controls (*p* = 0.008, and *p* = 0.053, respectively). The comparison between untreated patients and treated patients revealed differences in allele and genotype frequencies of *IL-10*_rs1800872 (*p* < 0.010), and untreated patients compared to the controls in the clinical group demonstrated differences in allele and genotype frequencies for *IL-10*_rs1800872 (*p* < 0.010), and in allele frequency for the *IL-13*_rs20541 variant (*p* = 0.030) (Table 2).

OR and RR values of gene frequencies between untreated patients and controls in the clinical group were also calculated (Supplementary Table S2).

This analysis showed that being a carrier of the minor allele of *IL-10*_rs1800896 has a potential protective effect, OR = 0.28 (CI_95%_ = 0.11–0.72, *p* = 0.010). In contrast, the genotype *IL-10*_rs1800872 TT in untreated patients with an OR = 4.36 (CI_95%_ = 1.23–15.54, *p* = 0.040) was linked to an increased PD risk, but being heterozygous for this variant has a protective effect (OR = 0.20; CI_95%_ = 0.05–0.78, *p* = 0.030) in controls. Further analysis of genetic variants of *IL-10* showed that the TT-TT diplotype was more frequent in patients than controls, yielding an OR = 3.64 (CI_95%_ = 0.99–13.84), RR = 1.66 (CI_95%_ = 1.03–2.68). Analyses of the remaining diplotypes and haplotypes did not show any significant results (Supplementary Table S2).

The genetic frequencies found were like those formerly documented in the *IL-10* [33], *IL-13* [34,35], and *IL-17A* genes [36,37] in an MM population (*p* > 0.050). Genetic frequencies were also compared to other populations in the gnomAD v.4.1.0 database. The allele frequency of the SNV *IL-10*_rs1800896 ranges from 0.053 in East Asians to 0.48 in Europeans; herein, the observed MM allelic frequency was comparable to that reported for Admixed American populations. The allele frequency of the *IL-10*_rs1800872 variant is 0.37 in East Asians to 0.77 in Europeans; the observed frequency in an MM population was 0.60, which is comparable to Africans/African Americans (*f* = 0.59). The *IL-13*_rs20541 variant is very frequent in all populations (0.54–0.81); our results showed a frequency close to that observed in Latin Americans (*f* = 0.43 vs. *f* = 0.51). The *IL-17A*_rs2275913 and rs8193036 variants exhibit highly variable allelic frequencies in the world’s populations (0.09 in Africans and 0.47 in Finns; 0.3 in East Asians to 0.79 in Middle Eastern populations), whereas their frequencies in MMs were 0.17 and 0.83, respectively.

### 3.3. Association Analysis of Genetic Variants and Clinical and Biochemical Parameters

The analysis of plasma concentrations of cytokines (baseline and one year after DRT) and inflammation indexes vs. the observed genotypes in patients and controls in the clinical group, under all inheritance models tested for each variant, revealed several differences (Table 3 and Supplementary Table S3). The genotypes of the *IL-10*_rs1800896 variant suggested that being a carrier of the minor allele C is a protective factor for inflammation; hence, the homozygous CC carriers exhibited the lowest inflammation indexes in the controls of the clinical group. In contrast, the TT genotype showed the highest NLI values in untreated PD patients vs. the TC genotype (2.26 ± 0.96 vs. 1.56 ± 0.20 *p* = 0.01), and similar findings were also seen in controls. The TT carriers of the *IL-10*_rs1800872 variant showed the highest SII indexes in patients (491.62 ± 216.80 vs. 337.34 ± 68.99 for TG carriers, *p* = 0.05) and controls. Interestingly, controls harboring the *IL-10*_rs1800872 GG genotype exhibited the lowest IL-10 levels after one year of DRT vs. individuals carrying TT + TG genotypes (3.73 ± 3.34 vs. 2.06 ± 0.68, *p* = 0.04) (Table 3 and Supplementary Table S3). A comparison of plasma IL-10 levels between patients who were carriers of the reference diplotype (TT-TT) and alternative diplotypes (2.88 vs. 3.57 *p* = 0.20), and alternative heterozygous/homozygous diplotypes (2.88 vs. 4.24 *p* = 0.17 and 2.88 vs. 5.52 *p* = 0.09, respectively), did not show differences among patients. This could be due to the disease per se, suggesting that genetic variants are not enough or sufficient to explain the inflammatory process in PD patients. Also, the comparison of patients carrying the diplotype TT-TT vs. TC-GG (2.22 ± 1.04 vs. 1.52 ± 0.12, *p* = 0.030) exhibited differences in the NLI inflammation marker. In contrast, there was a significant difference between patients and controls who were carriers of *IL-10* diplotype TT-TT (500.32 ± 231.23 vs. 273.89 ± 20.34, *p* ≤ 0.010). Moreover, in controls, there were significant differences in SII values between the reference and alternative haplotypes (i.e., TT-TT vs. TC-TG, 273.89 ± 20.34 vs. 576.28 ± 110.71, *p* ≤ 0.010) that were not observed in patients.

For the SNV *IL-13*_rs20541, the comparison of the AA vs. AG + GG genotypes with plasma IL-13 levels, between untreated patients and the clinical control group, was significant (5.13 ± 5.16 vs. 0.00, *p* = 0.029) (Table 3 and Supplementary Table S3). Regarding the *IL-17A*_rs8193036 variant, the patients carrying the TT genotype exhibited the lowest levels of the NLI value of inflammation compared to the other genotypes (TT vs. CC + CT; 1.68 ± 0.46 vs. 2.51 ± 1.04, *p* = 0.024).

### 3.4. Haplotype Analysis

**Figure 1:**
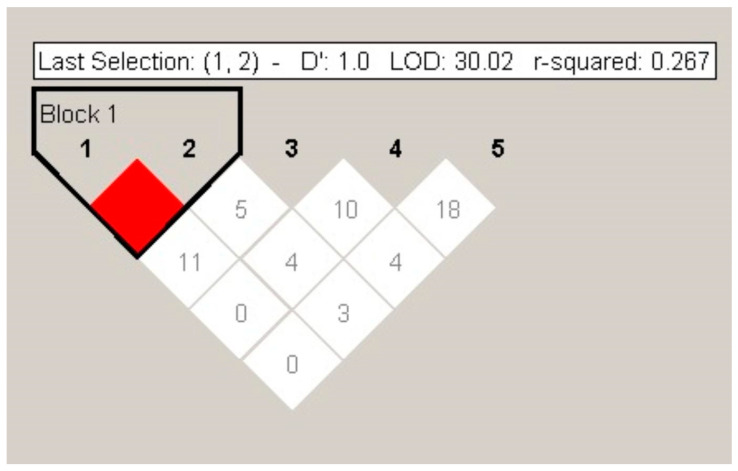
Haplotype block structure of *IL-10* rs1800896 and rs1800872. Both genetic variants are in linkage disequilibrium (LD). LOD, logarithm of the odds score. Dark red represents complete LD or stronger association between these two genetic markers, while lighter or white show weaker or no LD.

The haplotype analysis for the SNVs of *IL-10* studied is shown in Figure 1. Then, LD analysis revealed that *IL-10_*rs1800896 and *IL-10_*rs1800872 variants showed high LD (D′ = 1.0) with a weak association of r^2^ = 0.27. Four different haplotypes were inferred (TT, TG, TC, and CG) in our sample. Afterwards, the frequencies of the variants that were in LD were compared between patients and controls, but the results showed no differences: *f*(TT) = 0.404, *f*(TG) = 0.326, *f*(TC) = 0.21, and *f*(CG) = 0.270 in PD patients vs. *f*(TT) = 0.434, *f*(TG) = 0.295, *f*(TC) = 0.26, and *f*(CG) = 0.271 in controls (*p* > 0.050). Herein, the two *IL-17A* variants included did not present LD; therefore, no additional studies were performed (Supplementary Tables S2 and S3, Table 3).

**Figure 2:**
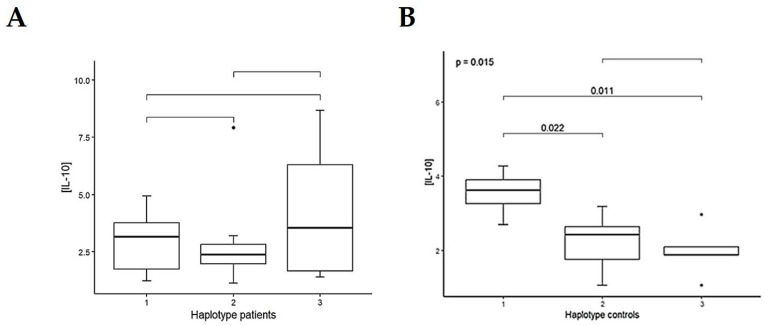
Box plots showing the plasma IL-10 levels according to its haplotypes, H1, H2, and H3 (see text), in (**A**) untreated PD patients in the clinical group and (**B**) controls in the clinical group. H1 was considered as the reference haplotype (T-T). *p*-values were calculated by an ANOVA test.

Then, the *IL-10* haplotype analysis showed differences in plasma IL-10 levels only in controls: H1 vs. H2, *p* = 0.022, and H1 vs. H3, *p* = 0.011, with an overall *p*-value = 0.015 (Figure 2, and Supplementary Table S2).

**Figure 3:**
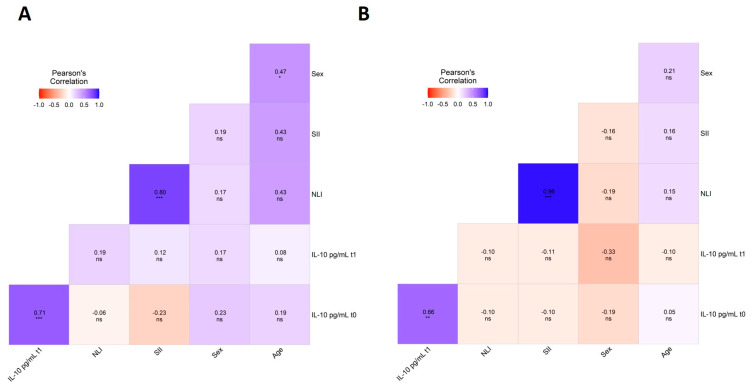
Multivariate correlation analysis between inflammatory indexes (NLI, SII) at the baseline, and plasma IL-10 levels at the baseline (t0) and one year after DRT (t1) in the PD patients of the clinical group (**A**). Multivariate analysis between NLI, SII, and IL-10 levels at t0 and t1 in controls of the clinical group (**B**). ns *p* ≥ 0.05; * *p* < 0.05; ** *p* < 0.01; *** *p* < 0.001.

Lastly, the multivariate analysis showed a highly significant positive correlation between cytokine IL-10 levels at the baseline and after one year of DRT in PD patients (r = 0.71, *p* < 0.001) and in controls (r = 0.66, *p* < 0.01) (Figure 3A,B). A positive correlation between inflammation indexes was observed in patients and controls of the clinical group (Figure 3A,B).

Promoter variants of pro- and anti-inflammatory cytokine genes *IL-17A* and *IL-10* were significantly associated with PD development in MM patients. Associations between genetic variants and clinical data (i.e., inflammation indexes and plasma levels of cytokines) were explored between untreated PD patients and controls in the clinical group (Table 3 and Supplementary Table S3).

There were some other associations between frequencies of the *IL-10* reference diplotypes and inflammation indexes in patients and controls, as follows: TT-TT was associated with higher SII values in patients vs. controls (500.32 ± 231.23 vs. 273.89 ± 20.34, *p* = 0.006), but TT-TT vs. TC-TG in controls exhibited lower SII values (273.89 ± 20.34 vs. 576.28 ± 110.71, *p* ≤ 0.001), while the comparisons of TT-TT vs. TC-GG diplotypes in patients showed elevated NLI values (2.22 ± 1.04 vs. 1.52 ± 0.12, *p* = 0.03).

## 4. Discussion

Chronic inflammation (in the nervous system or peripheral) plays a central role in PD risk, standing out in terms of the relevant inflammation in the progression of the disease. However, little is known about the impact of genetic variants of cytokines related to immune response in the development and progression of PD in MM patients. Herein, after determining the gene frequencies of functional variants in *IL-10*, *IL-13*, and *IL-17A*, we suggest that the *IL-17A*_rs8193036 variant may be associated with an increased risk for PD development. In addition, we observed that the *IL-10*_rs1800872 and *IL-10*_rs1800896 variants could be related to higher inflammation indexes, leading to an inflammatory profile and contributing to the severity of PD.

IL-10 is an anti-inflammatory cytokine that exerts a neuroprotective effect that is mediated through its receptor via the JAK/STAT3 pathway. This activation leads to a decrease in the cleavage of caspase-3 and caspase-9, which in turn reduces neuronal death in models of PD. IL-10 has been shown to promote increased primary neurite length in dopaminergic neurons [43]. Altogether, these effects support IL-10 as a key cytokine in suppressing inflammation and preventing neurodegeneration.

Functional genetic variants at positions c.-1082 (rs1800896), c.-819 (rs1800871), and c.-592 (rs1800872) in the *IL-10* promoter region are known to influence IL-10 production levels and are associated with various diseases of an inflammatory nature [38,44]. In the literature, the T allele of rs1800872 has been associated with increased serum of IL-10 levels [45], higher risks of cervical cancer development [46], and higher frequencies of severe and decreased outcomes in non-vaccinated COVID-19 patients [47]. In fact, it has been postulated that the ability of IL-10 to negatively regulate inflammation may be impaired in PD patients [48], and genetic variants may partly explain this.

Our results showed differences in allele and genotype frequencies of *IL-10*_rs1800872 between untreated patients and treated patients (*p* < 0.01) as well as controls of the clinical group (*p* < 0.01). This finding suggests that the TT genotype is a risk factor for PD with an OR = 4.36, whereas the TG genotype might be a protective factor with an OR = 0.20. The comparison of SII values in patient carriers of the TT vs. TG genotype (491.62 ± 216.8 vs. 337.34 ± 68.99, *p* = 0.05) also revealed a trend of higher levels of systemic inflammation in the TT genotype, in concordance with its role as a risk factor for PD.

The CC carriers of the *IL-10*_rs1800896 variant exhibited the lowest inflammation indexes in controls of the clinical group. In contrast, the TT genotype showed the highest NLI values in untreated PD patients vs. the TC genotype (2.26 ± 0.96 vs. 1.56 ± 0.20 *p* = 0.01), and similar findings were also seen in controls. This is in agreement with a report of the TT genotype being associated with a lower expression of IL-10 and the C allele being associated with higher IL-10 production [49]. The CC genotype has also been associated with a reduced risk of acute coronary disease in Mexican patients [50,51]. As has been previously reported [38], a high linkage disequilibrium (D′ = 1.0) between *IL-10_*rs1800896 and *IL-10_*rs1800872 variants was observed. The reference diplotype TT-TT was more frequent in PD untreated patients than controls (*p* = 0.02), increasing the risk for PD by nearly four times. As expected, the presence of the *IL-10* TT-TT reference diplotype was associated with higher NLI inflammation indexes in PD patients at the baseline (t0).

Some researchers have investigated a possible relationship between *IL-10* genetic variants and the potential risk of PD onset, with contradictory results. There are studies reporting no association [17,52], while other studies showed that *IL-10* promoter genetic variants are associated with PD risk and early onset [10,53]. In our study, the *IL-10* genetic variants included were associated with PD risk and higher indexes of inflammation. Also, controls in the clinical group exhibited differences in plasma IL-10 levels according to the stratified haplotype, showing higher plasma IL-10 levels in H1 than H2 > H3 haplotype carriers. However, this behavior was not observed in PD patients, thereby highlighting the possible involvement of IL-10 in the risk of PD onset.

IL-17 plays a dual role in PD, not only promoting inflammation but also directly contributing to neurodegeneration. Experimental models have demonstrated that IL-17 induces neuronal death via IL-17/IL-17R signaling and activation of the NF-κB pathway, whereas blocking IL-17 or its receptor markedly reduces neuronal death [7]. In this study, we included the *IL-17A*_rs2275913 and *IL-17A*_rs8193036 variants, which are in the promoter region of the gene. It has been reported that cells harboring the A allele (GA/AA genotypes) for the former variant secrete more IL-17A than cells negative for this allele [54,55,56]. In line with these reports, we observed higher plasma levels of IL-17A in AA > GA > GG carriers of *IL-17A*_rs2275913 in controls. Although non-significant, higher plasma levels of IL-17A were found in GA than GG PD patients. No associations were found with the *IL-17A*_rs2275913 variant. The genetic variant *IL-17A*_rs8193036 was found to be associated with an increased risk of developing PD with the lowest levels of this cytokine at the baseline. This functional SNV is in the promoter region of *IL-17A* and could regulate gene expression [28]. Its functionality has been observed in PBMCs from individuals carrying *IL-17A* rs8193036CC genotypes, who produced significantly lower amounts of IL-17A, and lower promoter transcription activities compared to rs8193036TT carriers [28]. In contrast, herein, carriers of the TT genotype showed the lowest NLI values (TT vs. CC, 1.68 ± 0.46 vs. 2.75 ± 1.38, *p* = 0.020) in the patients of the clinical group, indicating additional factors modulating this particular inflammation index. Genetic variation at the promoter region of the *IL-17A* gene would possibly enhance the binding of a transcription factor and increase production of IL-17A cytokines [57]. Moreover, it has been reported that the *IL-17A*_rs8193036 and *IL-17A* haplotypes are significantly associated with the risk of central obesity, hypertriglyceridemia, and metabolic syndrome in MM patients with premature coronary artery disease [36]; nonetheless, we did not observe LD for these variants in our sample.

Nevertheless, the above observed associations of *IL-10* and *IL-17A* genetic variants with the studied inflammatory indices should be acknowledged as early data, since they were obtained in a small cohort with uneven sample sizes.

There is evidence that genetic variants of *IL-13* confer a gain of function that may increase the risk of developing PD, by increasing cellular susceptibility to oxidative stress [58]. Moreover, IL-13 is mainly activated by Th2 cells, and a reasonable hypothesis is that the imbalance in the Th1/Th2 ratio could be involved in the pathogenesis of chronic diseases associated with age (e.g., PD) and be the result of dysfunction in the regulation of the immune system [59]. In this study, the comparison between untreated patients vs. treated patients revealed differences in the allele frequencies of the genetic variant *IL-13_*rs20541 (*p* = 0.03) (Table 2).

The combined activity of IL-10, IL-17A, and IL-13 is context-dependent, suggesting complex and sometimes contradictory roles, with some interactions exacerbating or dampening inflammation in PD. Researchers are currently studying whether inflammation itself is a cause of neurodegeneration or a response to an already-ongoing process that contributes to the development of PD. Previous work of our group reported that plasma IL-17A levels in untreated PD patients were significantly different to those in controls (*p* = 0.014), and then at 2 years post-DRT their Tc17 cell levels were increased [30]. Later, we found a positive correlation between mitochondrial DNA content and IL-17A levels in the untreated PD patients (r = 0.45, *p* < 0.05) included here [41]. Tc17 cells are responsible for producing cytokines IL-17A, IL-21, and IL-23. In the present study, our results suggest that the genetic variants of *IL-10*_rs1800872 and *IL-17A*_rs2275913, rs8193036 are related to altered cytokine profiles and may increase the risk of PD in MM patients. Furthermore, the positive correlation observed in plasma IL-10 levels at the baseline and after one year of DRT, exclusively for PD patients, indicates that the immune response remains persistently activated in patients, even after one year of treatment. In this context, our previous and present findings suggest a peripheral inflammatory profile in untreated PD patients; all these factors together might lead to oxidative stress and increase the production of pro-inflammatory cytokines in PD patients.

Our findings indicate that the *IL-10*_rs1800896, *IL-10*_rs1800872, and *IL-17A*_rs8193036 variants are closely linked to the inflammatory process. This is especially relevant in the immune context of PD, where there is an imbalance between anti-inflammatory Treg cells (the main producers of IL-10) and pro-inflammatory Th17 cells (the major producers of IL-17). Several reports have consistently shown a shift in CD4^+^ T cell populations, with an increased proportion of Th17 cells and a reduced proportion of Tregs. This imbalance correlates with greater disease severity [8,9,26]. Furthermore, the shift in the Th17/Treg balance toward Th17 during PD may contribute to neuroinflammation in PD patients.

Neurodegeneration and neuroinflammation are two intertwined mechanisms contributing to PD pathophysiology, and it is not clearly established if these processes differ according to disease duration [60]. Moreover, there is a study that characterized circulating biomarkers in PD patients with short and long disease duration (i.e., ≤5 or >5 years, respectively), and their results showed that a pro-inflammatory status may be observed in PD patients in the early phases of the disease, independently from age [60]. In this context, the involvement of inflammation in PD has been reported as maximized in the early disease stages and maintaining a chronic profile during the disease [61,62].

It has been proposed that immune cell ageing may synergize with a genetic predisposition background and environmental stressors to accelerate PD [4]. In these PD patients with genetic risk tied to the immune system, it would be important to prove anti-inflammatory therapies [63]. Further longitudinal studies should test this hypothesis. We tried to determine whether genetic predisposition added to pre-diagnostic levels of inflammatory biomarkers contribute to the risk and progression of PD. The studied genetic variants exhibit highly variable allelic frequencies in the world’s populations. Therefore, it is important to identify regional genetic patterns to better comprehend PD risk and develop targeted preventive strategies. The early detection of high-risk PD alleles could improve patient outcomes, reduce healthcare burden, and inform public health policies. In this context, a 4-year follow-up study performed in six Latin American countries revealed that PD patients have limited access to health services; thus, PD is associated with hospital admission, incident dependency, and all-cause mortality. This work also pointed out the need to identify new markers of diagnosis, prognosis, or evolution for this disorder [64].

We are aware of several limitations of our study, including the small sample size of participants with clinical data and an unbalanced sample of controls; thus, these limitations have hindered additional statistical analysis. In this sense, our findings should be interpreted with caution as preliminary data. Because the MM population has a distinct genetic background [65], the *IL-10* and *IL-17A* variant associations observed here should be explored in other populations. Future replication studies involving PD patients of diverse ancestries in larger, independent cohorts are required to validate the observed associations. Plasma cytokine levels can be influenced by environmental exposures and biological variables, which are non-related PD conditions. We analyzed plasma cytokine levels, which may not fully reflect changes in the central nervous system.

## 5. Conclusions

This study suggests that genetic variants in *IL-10* and *IL-17A* are related to the inflammatory process in MM patients with sporadic PD and may play a role in the risk and severity of PD. Future longitudinal research should delve deeper into genetic and environmental factors modulating inflammation as risk factors of developing and progression in PD patients.

## Figures and Tables

**Table 1:** Demographic, biochemical, and clinical characteristics of patients with Parkinson’s disease and healthy volunteers.

Characteristics	Treated PD Patients (n = 239)			Controls (n = 84)		
	Total (n = 239)	Male (n = 127)	Female (n = 112)	Total (n = 84)	Male (n = 38)	Female (n = 46)
Sex (%)	100	53.16	46.84	100	45.45	54.54
Age in years, mean ± SD (range)	59.15 ± 12.63 (23–88)	58.4 ± 12.24 (23–84)	60.05 ± 13.05 (29–88)	47.87 ± 18.54 (21–71)	45.21 ± 17.91 (21–69)	44.68 ± 18.79 (19–83)
	Untreated PD Patients in the Clinical Group			Controls in the Clinical Group		
	Total (n = 26)	Male (n = 16)	Female (n = 10)	Total (n = 21)	Male (n = 11)	Female (n = 10)
Sex (%)	100	61	39	100	52	48
Age in years, mean ± SD (range)	60.93 ± 10.60 (38–85)	59.56 ± 10.53 (38–85)	63.00 ± 10.82 (41–81)	55.73 ± 10.20 (34–81)	54.58 ± 8.39 (40–60)	57.00 ± 12.38 (34–81)
Weight (Kg), mean ± SD (range)	70.71 ± 13.13 (48–97)	75.82 ± 12.07 (54–97)	62.36 ± 10.55 (48–81)	69.21 ± 8.96 (54–90)	70.62 ± 7.17 (59–81.5)	67.52 ± 10.90 (54–90)
Height (cm), mean ± SD (range)	160.07 ± 11.29 (138–180)	166.11 ± 7.53 (150–180)	150.18 ± 9.36 (138–169)	159.77 ± 8.60 (143–176)	165.08 ± 6.17 (154–176)	153.4 ± 6.58 (143–167)
BMI, mean ± SD (range)	27.55 ± 4.74 (20.61–37.4)	27.26 ± 13.02 (26.61–33.20)	28.02 ± 6.36 (21.69–37.40)	26.85± 3.89 (20.7–36.4)	26.13 ± 3.21 (20.7–32.91)	27.72 ± 4.60 (22.5–36.4)
Cholesterol (mg/dL), mean ± SD (range)	189.24 ± 34.35 (122.1–266.6)	176.36 ± 32.75 (122.10–236.15)	209.87 ± 26.88 (185.18–266.60)	217.35 ± 55.95 (139.94–377)	213.07 ± 49.05 (139.94–309)	222.49 ± 65.65 (154–377)
Triglycerides (mg/dL), mean ± SD (range)	140.23 ± 55.02 (47.35–293.4)	127.17 ± 46.25 (47.35–216.6)	162.42 ± 63.83 (77.5–293.4)	162.17 ± 63.02 (74.5–318.52)	166.11 ± 73.31 (89.27–318.52)	157.45 ± 51.51 (74.5–229.1)
Smokers, n (%)	3 (10)	3 (16.7)	0	9 (40.9)	8 (66.7)	1 (10)
Alcohol consumers, n (%)	10 (33.3)	9 (50)	1 (8.3)	10 (45.5)	8 (66.7)	2 (20)
Uric acid (mg/dL), mean ± SD (range)	5.77 ± 1.37 (3.7–9.18)	6.40 ± 1.30 (4.51–9.18)	4.70 ± 0.60 (3.7–5.8)	6.13 ± 1.50 (4.01–9.35)	6.69 ± 1.46 (4.8–9.35)	5.47 ± 1.31 (4.01–7.95)
IL-10 (pg/mL), mean ± SD (range)	2.95 ± 2.02 (0–8.67)	2.44 ± 1.79 (0–7.91)	3.71 ± 2.18 (1.44–8.67)	2.68 ± 2.54 (0.08–13.02)	3.12 ± 3.27 (0.41–13.02)	2.17 ± 1.20 (0.08–3.78)
IL-13 (pg/mL), mean ± SD (range)	4.38 ± 7.34 (0–31.67)	3.74 ± 5.30 (0–17.10)	5.86 ± 8.99 (0–31.67)	0	0	0
IL-17A (pg/mL), mean ± SD (range)	1341.87 ± 1365.9 (73.4–3904.4)	1363.9 ± 1424.55 (73.4–3904.4)	1308.81 ± 1334.30 (77.4–3888.4)	2250.71 ± 1365.93 (6.4–3937.4)	2354.57 ± 1370.27 (6.4–3937.4)	2126.1 ± 1423.70 (515.4–3898.4)
NLI, mean ± SD (range)	1.68 ± 0.46 (0.71–3.05)	1.71 ± 0.50 (0.71–3.05)	1.63 ± 0.41 (1.05–2.17)	1.99 ± 1.56 (0.53–8.44)	1.81 ± 0.58 (1.27–2.96)	2.26 ± 2.22 (0.53–8.44)
SII, mean ± SD (range)	399.31 ± 176.35 (78.1–883.89)	387.82 ± 154.33 (78.1–671.04)	418.11 ± 214.38 (203.49–883.89)	492.84 ± 454.88 (108.36–2447.31)	415.91 ± 186.73 (108.36–777.02)	554.47 ± 688.78 (110.76–2447.31)
Clinical Scales for the Staging of the Functional Disability of Patients						
HOEN and YAHR scale, median (range)	2 (1–4)	2 (1–3)	2 (1–4)	NA	NA	NA
MDS-UPDRS I, mode (range)	4 (0–6)	4 (0–6)	4 (0–4)	NA	NA	NA
MDS-UPDRS II, mode (range)	10 (3–25)	10 (3–25)	15 (5–25)	NA	NA	NA
MDS-UPDRS III, mode (range)	38 (4–72)	38 (4–60.5)	13 (13–72)	NA	NA	NA
MDS-UPDRS IV	0	0	0	NA	NA	NA
MDS-UPDRS Total, mode (range)	52 (8–93)	47 (8–88)	52 (21–93)	NA	NA	NA
Schwab and England scale, mode (range)	90 (20–100)	90 (50–100)	90 (20–90)	100 (90–100)	100 (100–100)	100 (90–100)
Beck’s Depression Inventory, mode (range)	10 (0–30)	10 (0–23)	10 (2–30)	2.5 (0–21)	1.5 (0–13)	7 (0–21)

BMI, body mass index, was calculated using a participant’s height and weight. NA: not applicable. NLI: neutrophil/lymphocyte inflammation index. SII = systemic inflammatory index. MDS-UPDRS I: Non-Motor Experiences of Daily Living. MDS-UPDRS II: Motor Experiences of Daily Living. MDS-UPDRS III: Motor Examination. MDS-UPDRS IV: Motor Complications. Schwab and England (Activities of Daily Living) scale. Categorical characteristics include percentages; continuous variables are presented as means with standard deviations and ranges. Significant comparisons (*p* < 0.05) between the study groups are explained in the Results section.

**Table 2:** Allele and genotype frequency distribution for genetic variants in patients with Parkinson’s disease and healthy volunteers.

Genetic Characteristic			Total Group of PD Patients (n = 239 and 478 Alleles)			Total Group of Controls (n = 84 and 168 Alleles)			*p*-Values		
Gene	Variant ID	Genotype/Allele	n	Frequency	CI_95%_	n	Frequency	CI_95%_	*p* ^1^	*p* ^2^	*p* ^3^
*IL-10*	rs1800896	TT	118	0.54	0.43–0.56	42	0.50	0.39–0.61	1.00	0.22	0.18
		TC	93	0.39	0.33–0.45	34	0.40	0.30–0.52	0.79	0.83	0.65
		CC	18	0.08	0.05–0.12	8	0.10	0.04–0.18	0.64	0.24	0.19
		T	349	0.73	0.69–0.77	118	0.70	0.63–0.77	0.48	0.18	0.14
		C	129	0.27	0.23–0.31	50	0.30	0.23–0.37			
	rs1800872	TT	45	0.19	0.14–0.24	13	0.15	0.09–0.25	0.62	**<0.01**	**<0.01**
		TG	103	0.43	0.37–0.50	46	0.55	0.44–0.66	0.07	**<0.01**	**<0.01**
		GG	91	0.38	0.32–0.45	25	0.30	0.20–0.41	0.18	0.29	1.00
		T	193	0.40	0.36–0.45	72	0.43	0.35–0.51	0.58	**<0.01**	**<0.01**
		G	285	0.60	0.55–0.64	96	0.57	0.49–0.65			
*IL-13*	rs20541	AA	82	0.34	0.28–0.41	24	0.29	0.19–0.39	0.35	0.13	0.06
		AG	110	0.46	0.40–0.53	42	0.50	0.39–0.61	0.61	0.84	0.51
		GG	47	0.20	0.15–0.25	18	0.21	0.13–0.32	0.75	0.18	0.15
		A	278	0.58	0.54–0.63	90	0.54	0.46–0.61	0.32	**0.05**	**0.03**
		G	210	0.42	0.39–0.49	78	0.46	0.39–0.54			
*IL-17A*	rs2275913	GG	162	0.68	0.61–0.74	58	0.69	0.58–0.79	0.89	0.83	0.81
		GA	66	0.28	0.22–0.34	23	0.27	0.18–0.38	1.00	0.82	0.80
		AA	11	0.04	0.02–0.08	3	0.04	0.01–0.10	1.00	1.00	1.00
		G	390	0.82	0.78–0.85	139	0.83	0.76–0.88	0.82	0.85	0.84
		A	88	0.18	0.15–0.22	29	0.17	0.12–0.24			
	rs8193036	CC	33	0.14	0.10–0.19	3	0.04	0.01–0.10	**<0.01**	1.00	0.16
		CT	70	0.29	0.24–0.36	23	0.27	0.18–0.38	0.78	0.65	0.47
		TT	136	0.57	0.50–0.63	58	0.69	0.58–0.79	0.05	0.87	0.36
		C	136	0.28	0.24–0.33	29	0.17	0.12–0.24	**<0.01**	1.00	0.08
		T	342	0.72	0.67–0.76	139	0.83	0.76–0.88			

Variant ID, from the dbSNP database. These identifiers are assigned to unique variations within a reference genome. PD: Parkinson’s disease. n: number. CI: confidence interval. Untreated patients (n = 26) and controls (n = 21) in the clinical group. Significant *p*-values are shown in bold. *p* ^1^ = PD patients (n = 239) vs. controls (n = 84). *p* ^2^ = PD patients (n = 239) vs. untreated patients (n = 26). *p* ^3^ = Untreated patients (n = 26) vs. controls in the clinical group (n = 21). *p*-values were calculated by Fisher’s exact test.

**Table 3:** Analysis of genotypes, inflammation indexes, and plasma levels of cytokines between untreated PD patients and controls in the clinical group.

Genetic Characteristic			Untreated PD Patients in the Clinical Group (n = 26)					Controls in the Clinical Group (n = 21)				
Gene	Variant ID	Genotype	n	NLI	SII	^†^ IL Plasma Levels	^‡^ IL Plasma Levels	n	NLI	SII	^†^ IL Plasma Levels	^‡^ IL Plasma Levels
*IL-10*	rs1800896	TT	17	**2.26** **(0.96)**	479.57 (203.30)	2.79 (1.84)	2.87 (1.97)	8	2.2 (2.51)	538.62 (775.43)	2.55 (1.37)	2.41 (0.77)
		TC	9	1.56 (0.20)	409.23 (181.18)	3.72 (2.58)	3.51 (2.23)	8	2.08 (0.56)	540.96 (114.32)	3.31 (4.059)	4.39 (4.01)
		CC	0	---	---	---	—	5	1.2 (0.44)	349.33 (152.05)	1.98 (0.68)	2.24 (0.58)
	rs1800872	TT	15	2.12 (0.98)	**491.62** **(216.80)**	2.84 (1.08)	2.99 (1.84)	5	2.79 (3.16)	743.09 (955.73)	2.86 (1.65)	2.46 (0.86)
		TG	4	1.79 (0.33)	337.34 (68.99)	7.913	5.47	10	1.93 (0.60)	449.77 (196.00)	1.96 (0.94)	4.52 (3.91)
		GG	7	2.07 (0.85)	437.23 (173.47)	3.64 (3.262)	2.64 (2.57)	6	1.27 (0.39)	361.71 (139.34)	3.82 (4.55)	2.06 (0.68)
	Diplotype	TT-TT	12	2.22 (1.04)	500.32 (231.23)	2.88 (1.18)	3.02 (2.02)	4	3.10 (3.6)	**273.89** **(20.34)**	**3.55** **(0.66)**	3.19
		TT-TG	2	1.79	337.34	4.79	4.027	4	1.38 (0.23)	259.99 (121.59)	1.55 (1.12)	2.03 (0.63)
		TT-GG	3	2.9	518.08	1.13 (1.13)	1.58 (1.68)	0	NA	NA	NA	NA
		TC-TT	3	1.62	448.10	2.68 (0.44)	2.81	1	1.54	446.45	0.08	1.74
		TC-TG	2	ND	ND	1.66	ND	6	2.29 (0.47)	**576.28** **(110.71)**	2.22 (0.78)	3.87 (3.48)
		TC-GG	4	**1.52** **(0.12)**	383.32 (217.95)	5.52 (3.07)	4.22	1	1.37	423.61	13.02	10.78
		CC-GG	0	NA	NA	NA	NA	5	1.25 (0.44)	349.33 (152.05)	1.978 (0.68)	2.24 (0.58)
*IL-13*	rs20541	AA	13	1.76 (0.49)	356.92 (103.36)	**5.13** **(5.16)**	**0**	8	2.43 (2.45)	667.99 (725.51)	0	10.42 (16.14)
		AG	11	2.19 (0.82)	465.45 (99.94)	0	3.556 (7.951)	8	1.60 (1.39)	385.54 (195.94)	0	15.54 (22.19)
		GG	2	0.98	320.59	0	0	5	1.73 (0.94)	391.03 (217.12)	0	0
*IL-17A*	rs2275913	GG	17	2.08 (0.87)	516.03 (142.19)	1218.34 (1382.52)	1741.95 (1119.02)	2	1.41 (0.11)	323.90 (12.48)	3472.9 (522.55)	ND
		GA	8	2.12 (1.01)	446.78 (221.42)	1932.28 (1191.93)	1446.14 (1766.87)	6	1.71 (0.33)	444.97 (89.57)	1494.23 (1257.09)	ND
		AA	1	1.61	233.36	383.4	585.40	13	2.14 (2.01)	543.52 (604.62)	2412.55 (1421.42)	ND
	rs8193036	CC	3	2.75 (1.38)	516.51 (192.01)	1267.07 (661.61)	2294.73 (1590.42)	2	1.77 (0.65)	439.43 (102.58)	2269.4 (1854.03)	ND
		CT	9	2.39 (1.06)	523.60 (285.52)	1456.29 (1797.25)	1706.57 (1738.23)	5	1.53 (0.41)	382.43 (182.24)	1066.8 (1016.43)	ND
		TT	14	**1.68** **(0.46)**	406.91 (126.26)	1381.11 (1186.82)	1274.62 (1050.33)	14	2.12 (1.92)	542.3 (574.26)	2671.54 (1304.51)	ND

Variant ID, from the dbSNP database. These identifiers are assigned to unique variations within a reference genome. PD: Parkinson’s disease. n: number. CI: confidence interval. NLI: neutrophil–lymphocyte index, and SII: systemic immune-inflammation index; both were used to evaluate inflammation. Untreated patients (n = 26) and controls (n = 21) in the clinical group. DRT: dopaminergic replacement therapy. NA: not applicable. ND: not determined. Values in bold showed association (see details in Supplementary Table S3 and text). Mean values are accompanied by the standard deviation value in parentheses (±SD). ^†^ IL plasma levels [pg/mL] at the baseline, before treatment (t0). ^‡^ IL plasma levels [pg/mL] after one year of DRT. *p*-values were calculated by the student’s *t*-test.

## Data Availability

The data presented in this study are available on request from the corresponding author due to ethical issues.

## Supplementary Materials

The following supporting information can be downloaded at: https://www.mdpi.com/article/10.3390/life15091480/s1, Table S1: Characteristics of the genetic variants included in the study; Table S2: Odds ratio and relative risk values of gene frequencies between untreated patients and controls in the clinical group; Table S3: Association between genetic variants of cytokines (genotype and haplotype) and IL-10 plasma levels, NLI, and SII in patients and controls of the clinical group. References [66,67,68,69,70,71,72,73] are cited in the supplementary materials.

## References

[1] Kouli A., Torsney K.M., Kuan W.L., Stoker T.B., Greenland J.C. (2018). Parkinson’s Disease: Etiology, Neuropathology, and Pathogenesis. Parkinson’s Disease: Pathogenesis and Clinical Aspects.

[2] Lees A.J., Hardy J., Revesz T. (2009). Parkinson’s disease. Lancet.

[3] Pajares M., IRojo A., Manda G., Boscá L., Cuadrado A. (2020). Inflammation in Parkinson’s Disease: Mechanisms and Therapeutic Implications. Cells.

[4] Tansey M.G., Wallings R.L., Houser M.C., Herrick M.K., Keating C.E., Joers V. (2022). Inflammation and immune dysfunction in Parkinson disease. Nat. Rev. Immunol..

[5] Li C., Ke B., Chen J., Xiao Y., Wang S., Jiang R., Zheng X., Lin J., Huang J., Shang H. (2024). Systemic inflammation and risk of Parkinson’s disease: A prospective cohort study and genetic analysis. Brain Behav. Immun..

[6] Liu T.W., Chen C.M., Chang K.H. (2022). Biomarker of Neuroinflammation in Parkinson’s Disease. Int. J. Mol. Sci..

[7] Sommer A., Marxreiter F., Krach F., Fadler T., Grosch J., Maroni M., Graef D., Eberhardt E., Riemenschneider M.J., Yeo G.W. (2019). Th17 Lymphocytes Induce Neuronal Cell Death in a Human iPSC-Based Model of Parkinson’s Disease. Cell Stem Cell.

[8] Chen Y., Qi B., Xu W., Ma B., Li L., Chen Q., Qian W., Liu X., Qu H. (2015). Clinical correlation of peripheral CD4^+^-cell sub-sets, their imbalance and Parkinson’s disease. Mol. Med. Rep..

[9] Kustrimovic N., Comi C., Magistrelli L., Rasini E., Legnaro M., Bombelli R., Aleksic I., Blandini F., Minafra B., Riboldazzi G. (2018). Parkinson’s disease patients have a complex phenotypic and functional Th1 bias: Cross-sectional studies of CD4^+^ Th1/Th2/T17 and Treg in drug-naïve and drug-treated patients. J. Neuroinflamm..

[10] Qin X.Y., Zhang S.P., Cao C., Loh Y.P., Cheng Y. (2016). Aberrations in Peripheral Inflammatory Cytokine Levels in Parkinson Disease: A Systematic Review and Meta-analysis. JAMA Neurol..

[11] Koziorowski D., Tomasiuk R., Szlufik S., Friedman A. (2012). Inflammatory cytokines and NT-proCNP in Parkinson’s disease patients. Cytokine.

[12] Rota E., Bellone G., Rocca P., Bergamasco B., Emanuelli G., Ferrero P. (2006). Increased intrathecal TGF-beta1, but not IL-12, IFN-gamma and IL-10 levels in Alzheimer’s disease patients. Neurol. Sci. Off. J. Ital. Neurol. Soc. Ital. Soc. Clin. Neurophysiol..

[13] Bialecka M., Klodowska-Duda G., Kurzawski M., Slawek J., Opala G., Bialecki P., Safranow K., Droździk M. (2007). Interleukin-10 gene polymorphism in Parkinson’s disease patients. Arch. Med. Res..

[14] Bialecka M., Klodowska-Duda G., Kurzawski M., Slawek J., Gorzkowska A., Opala G., Bialecki P., Sagan L., Droździk M. (2008). Interleukin-10 (IL10) and tumor necrosis factor alpha (TNF) gene polymorphisms in Parkinson’s disease patients. Park. Relat. Disord..

[15] Li D., He Q., Li R., Xu X., Chen B., Xie A. (2012). Interleukin-10 promoter polymorphisms in Chinese patients with Parkinson’s disease. Neurosci. Lett..

[16] Shu L., Liang D., Pan H., Xu Q., Yan X., Tang B., Sun Q. (2018). Gastrointestinal Dysfunctions Are Associated with *IL-10* Variants in Parkinson’s Disease. Park. Dis..

[17] Pascale E., Passarelli E., Purcaro C., Vestri A.R., Fakeri A., Guglielmi R., Passarelli F., Meco G. (2011). Lack of association between IL-1β, TNF-α, and IL-10 gene polymorphisms and sporadic Parkinson’s disease in an Italian cohort. Acta Neurol. Scand..

[18] Jin J., Wu P., Li W., Shi J., Chen J., Li R., Tan S. (2014). Interleukin-10-1082A/G and -592C/A polymorphisms with risk of Parkinson’s disease: A meta-analysis. Int. J. Neurosci..

[19] Liu Z., Guo J., Wang Y., Li K., Kang J., Wei Y., Sun Q., Xu Q., Xu C., Yan X. (2016). Lack of association between IL-10 and IL-18 gene promoter polymorphisms and Parkinson’s disease with cognitive impairment in a Chinese population. Sci. Rep..

[20] Tong Y., Jiang S., Cai L., Guan X., Hou S., Wang Z., Lu Q., Liu J. (2018). Identification of Functional Genetic Polymorphisms at IL-10 Promoter Region and their Association with Risk of Ischemic Stroke in Chinese Han Population. J. Nutr. Health Aging.

[21] Sun X., Gu R., Bai J. (2024). Differentiation and regulation of CD4^+^ T cell subsets in Parkinson’s disease. Cell. Mol. Life Sci. CMLS.

[22] Mori S., Maher P., Conti B. (2016). Neuroimmunology of the Interleukins 13 and 4. Brain Sci..

[23] Liu Z., Huang Y., Cao B.B., Qiu Y.H., Peng Y.P. (2017). Th17 Cells Induce Dopaminergic Neuronal Death via LFA-1/ICAM-1 Interaction in a Mouse Model of Parkinson’s Disease. Mol. Neurobiol..

[24] Liu Z., Qiu A.W., Huang Y., Yang Y., Chen J.N., Gu T.T., Cao B.B., Qiu Y.H., Peng Y.P. (2019). IL-17A exacerbates neuroinflammation and neurodegeneration by activating microglia in rodent models of Parkinson’s disease. Brain Behav. Immun..

[25] Chen J., Liu X., Zhong Y. (2020). Interleukin-17A: The Key Cytokine in Neurodegenerative Diseases. Front. Aging Neurosci..

[26] Álvarez-Luquín D.D., Arce-Sillas A., Leyva-Hernández J., Sevilla-Reyes E., Boll M.C., Montes-Moratilla E., Vivas-Almazán V., Pérez-Correa C., Rodríguez-Ortiz U., Espinoza-Cárdenas R. (2019). Regulatory impairment in untreated Parkinson’s disease is not restricted to Tregs: Other regulatory populations are also involved. J. Neuroinflamm..

[27] Rocha N.P., Assis F., Scalzo P.L., Vieira É.L.M., Barbosa I.G., de Souza M.S., Christo P.P., Reis H.J., Teixeira A.L. (2018). Reduced Activated T Lymphocytes (CD4^+^CD25^+^) and Plasma Levels of Cytokines in Parkinson’s Disease. Mol. Neurobiol..

[28] Wang W., Deng G., Zhang G., Yu Z., Yang F., Chen J., Cai Y., Werz O., Chen X. (2020). Genetic polymorphism rs8193036 of IL17A is associated with increased susceptibility to pulmonary tuberculosis in Chinese Han population. Cytokine.

[29] Cui H., Wang N., Li H., Bian Y., Wen W., Kong X., Wang F. (2024). The dynamic shifts of IL-10-producing Th17 and IL-17-producing Treg in health and disease: A crosstalk between ancient “Yin-Yang” theory and modern immunology. Cell Commun. Signal. CCS.

[30] Newcomb D.C., Boswell M.G., Huckabee M.M., Goleniewska K., Dulek D.E., Reiss S., Lukacs N.W., Kolls J.K., Peebles R.S. (2012). IL-13 regulates Th17 secretion of IL-17A in an IL-10-dependent manner. J. Immunol..

[31] Álvarez-Luquín D.D., Guevara-Salinas A., Arce-Sillas A., Espinosa-Cárdenas R., Leyva-Hernández J., Montes-Moratilla E.U., Adalid-Peralta L. (2021). Increased Tc17 cell levels and imbalance of naïve/effector immune response in Parkinson’s disease patients in a two-year follow-up: A case control study. J. Transl. Med..

[32] Zhao Y., Zhang X., Guo N., Tian D., Zhang C., Mu C., Han C., Zhu R., Zhang J., Liu X. (2022). Genetically Predicted Levels of Circulating Inflammatory Cytokines and the Risk and Age at Onset of Parkinson’s Disease: A Two-Sample Mendelian Randomization Study. Front. Aging Neurosci..

[33] Vargas-Alarcon G., Ramírez-Bello J., Juárez-Cedillo T., Ramírez-Fuentes S., Carrillo-Sánchez S., Fragoso J.M. (2012). Distribution of the IL-1RN, IL-6, IL-10, INF-γ, and TNF-α Gene Polymorphisms in the Mexican Population. Genet. Test. Mol. Biomark..

[34] Villarreal-Martínez A., Gallardo-Blanco H., Cerda-Flores R., Torres-Muñoz I., Gómez-Flores M., Salas-Alanís J., Ocampo-Candiani J., Martínez-Garza L. (2016). Candidate gene polymorphisms and risk of psoriasis: A pilot study. Exp. Ther. Med..

[35] Ambrocio-Ortiz E., Galicia-Negrete G., Pérez-Rubio G., Escobar-Morales A.J., Abarca-Rojano E., Del Angel-Pablo A.D., Castillejos-López M.D.J., Falfán-Valencia R. (2020). Single Nucleotide and Copy-Number Variants in IL4 and IL13 Are Not Associated with Asthma Susceptibility or Inflammatory Markers: A Case-Control Study in a Mexican-Mestizo Population. Diagnostics.

[36] Vargas-Alarcón G., Angeles-Martínez J., Villarreal-Molina T., Alvarez-León E., Posadas-Sánchez R., Cardoso-Saldaña G., Ramírez-Bello J., Pérez-Hernández N., Juárez-Rojas J.G., Rodríguez-Pérez J.M. (2015). Interleukin-17A gene haplotypes are associated with risk of premature coronary artery disease in Mexican patients from the Genetics of Atherosclerotic Disease (GEA) study. PLoS ONE.

[37] Ponce-Gallegos M.A., Pérez-Rubio G., Ambrocio-Ortiz E., Partida-Zavala N., Hernández-Zenteno R., Flores-Trujillo F., García-Gómez L., Hernández-Pérez A., Ramírez-Venegas A., Falfán-Valencia R. (2020). Genetic variants in IL17A and serum levels of IL-17A are associated with COPD related to tobacco smoking and biomass burning. Sci. Rep..

[38] Chavarria-Buenrostro L.E., Hernandez-Bello J., Muñoz-Valle J.F., Macias-Barragan J., Hernandez-Carrillo L.B., Topete-Reyes J.F., Parra-Michel R., Ramirez-Dueñas M.G., Sanchez-Hernandez P.E., Pereira-Suarez A.L. (2019). IL10 haplotypes are associated with diabetic nephropathy susceptibility in patients from western Mexico. J. Clin. Lab. Anal..

[39] Postuma R.B., Berg D., Stern M., Poewe W., Olanow C.W., Oertel W., Obeso J., Marek K., Litvan I., Lang A.E. (2015). MDS clinical diagnostic criteria for Parkinson’s disease. Mov. Disord. Off. J. Mov. Disord. Soc..

[40] Barrett J.C., Fry B., Maller J., Daly M.J. (2005). Haploview: Analysis and visualization of LD and haplotype maps. Bioinformatics.

[41] Ortega-Vázquez A., Sánchez-Badajos S., Ramírez-García M.Á., Alvarez-Luquín D., López-López M., Adalid-Peralta L.V., Monroy-Jaramillo N. (2023). Longitudinal Changes in Mitochondrial DNA Copy Number and Telomere Length in Patients with Parkinson’s Disease. Genes.

[42] Wickham H., Chang W., Henry L., Pedersen T.L., Takahashi K., Wilke C., Woo K., Yutani H., Dunnington D., van den Brand T. (2024). ggplot2: Create Elegant Data Visualisations Using the Grammar of Graphics [Internet]. https://cran.r-project.org/web/packages/ggplot2/index.html.

[43] Guevara-Salinas A., Netzahualcoyotzi C., Álvarez-Luquín D.D., Pérez-Figueroa E., Sevilla-Reyes E.E., Castellanos-Barba C., Vega-Ángeles V.T., Terán-Dávila E., Estudillo E., Velasco I. (2024). Treating activated regulatory T cells with pramipexole protects human dopaminergic neurons from 6-OHDA-induced degeneration. CNS Neurosci. Ther..

[44] Suárez A., Castro P., Alonso R., Mozo L., Gutiérrez C. (2003). Interindividual variations in constitutive interleukin-10 messenger RNA and protein levels and their association with genetic polymorphisms. Transplantation.

[45] Pavón-Romero G.F., Pérez-Rubio G., Ramírez-Jiménez F., Ambrocio-Ortiz E., Merino-Camacho C.R., Falfán-Valencia R., Teran L.M. (2020). *IL10* rs1800872 Is Associated with Non-Steroidal Anti-Inflammatory Drugs Exacerbated Respiratory Disease in Mexican-Mestizo Patients. Biomolecules.

[46] Pasvenskaite A., Liutkeviciene R., Gedvilaite G., Vilkeviciute A., Liutkevicius V., Uloza V. (2021). Impact of *IL-10* Promoter Polymorphisms and IL-10 Serum Levels on Advanced Laryngeal Squamous Cell Carcinoma and Survival Rate. Cancer Genom. Proteom..

[47] Martínez-Gómez L.E., Oropeza-Vélez C.I., Almonte-Becerril M., Chavez-Galan L., Martinez-Armenta C., Vidal-Vázquez R.P., Ramírez-Hinojosa J.P., Vázquez-Cárdenas P., Gómez-Martín D., Vargas-Alarcón G. (2025). Association of pro-inflammatory and anti-inflammatory cytokine polymorphisms with COVID-19 severity in unvaccinated patients. Front. Immunol..

[48] Dzamko N. (2023). Cytokine activity in Parkinson’s disease. Neuronal Signal..

[49] Liu N., Lu H., Tao F., Guo T., Liu C., Cui B., Ning G. (2011). An association of interleukin-10 gene polymorphisms with Graves’ disease in two Chinese populations. Endocrine.

[50] Posadas-Sánchez R., Angeles-Martínez J., Pérez-Hernández N., Rodríguez-Pérez J.M., López-Bautista F., Flores-Dominguez C., Fragoso J.M., Posadas-Romero C., Vargas-Alarcón G. (2018). The IL-10-1082 (rs1800896) G allele is associated with a decreased risk of developing premature coronary artery disease and some IL-10 polymorphisms were associated with clinical and metabolic parameters. The GEA study. Cytokine.

[51] Garcia-Garduño T.C., Padilla-Gutiérrez J.R., Aceves-Ramírez M., Parra-Reyna B., Flores-Salinas H.E., Valdes-Alvarado E., Becerra-Loaiza D.S., Quintero-Ramos A., Roa-Bruzón I.Y., de la Cruz A. (2024). IL10 promoter variants are associated with gene expression but they are not markers of susceptibility to acute coronary syndrome. Sci. Rep..

[52] Chu K., Zhou X., Luo B.Y. (2012). Cytokine gene polymorphisms and Parkinson’s disease: A meta-analysis. Can. J. Neurol. Sci..

[53] Porro C., Cianciulli A., Panaro M.A. (2020). The Regulatory Role of IL-10 in Neurodegenerative Diseases. Biomolecules.

[54] Ruddy M.J., Wong G.C., Liu X.K., Yamamoto H., Kasayama S., Kirkwood K.L., Gaffen S.L. (2004). Functional cooperation between interleukin-17 and tumor necrosis factor-alpha is mediated by CCAAT/enhancer-binding protein family members. J. Biol. Chem..

[55] Huang H.T., Lu Y.L., Wang R., Qin H.M., Wang C.F., Wang J.L., Xiang Y., Guo J., Lan Y., Wei Y.S. (2017). The association of IL-17A polymorphisms with IL-17A serum levels and risk of ischemic stroke. Oncotarget.

[56] Tang H., Pei H., Xia Q., Tang Y., Huang J., Huang J., Pei F. (2018). Role of gene polymorphisms/haplotypes and serum levels of interleukin-17A in susceptibility to viral myocarditis. Exp. Mol. Pathol..

[57] Lang X., Liu W., Hou Y., Zhao W., Yang X., Chen L., Yan Q., Cheng W. (2021). IL-17A polymorphism (rs2275913) and levels are associated with preeclampsia pathogenesis in Chinese patients. BMC Med. Genom..

[58] Aguirre C.A., Concetta Morale M., Peng Q., Sanchez-Alavez M., Cintrón-Colón R., Feng K., Fazelpour S., Maher P., Conti B. (2020). Two single nucleotide polymorphisms in IL13 and IL13RA1 from individuals with idiopathic Parkinson’s disease increase cellular susceptibility to oxidative stress. Brain Behav. Immun..

[59] Alvarez-Rodríguez L., López-Hoyos M., Carrasco-Marín E., Mata C., Calvo-Alén J., Aurrecoechea E., Blanco R., Ruiz T., Muñoz Cacho P., Villa I. (2012). Analysis of the rs20541 (R130Q) polymorphism in the IL-13 gene in patients with elderly-associated chronic inflammatory diseases. Reumatol. Clin..

[60] Di Lazzaro G., Picca A., Boldrini S., Bove F., Marzetti E., Petracca M., Piano C., Bentivoglio A.R., Calabresi P. (2024). Differential profiles of serum cytokines in Parkinson’s disease according to disease duration. Neurobiol. Dis..

[61] Kouli A., Williams-Gray C.H. (2020). Timing Is Everything: The T-Cell Response to α-Synuclein Is Maximal in Early Parkinson’s. Mov. Disord. Off. J. Mov. Disord. Soc..

[62] Lindestam Arlehamn C.S., Dhanwani R., Pham J., Kuan R., Frazier A., Rezende Dutra J., Phillips E., Mallal S., Roederer M., Marder K.S. (2020). α-Synuclein-specific T cell reactivity is associated with preclinical and early Parkinson’s disease. Nat. Commun..

[63] Wang Q., Liu Y., Zhou J. (2015). Neuroinflammation in Parkinson’s disease and its potential as therapeutic target. Transl. Neurodegener..

[64] Kim D.J., Rodriguez-Salgado A.M., Llibre-Rodriguez J.J., Acosta I., Sosa A.L., Acosta D., Jimenez-Velasquez I.Z., Guerra M., Salas A., Jeyachandran C. (2023). Burden of Parkinsonism and Parkinson’s Disease on Health Service Use and Outcomes in Latin America. J. Park. Dis..

[65] Juárez-Cedillo T., Zuñiga J., Acuña-Alonzo V., Pérez-Hernández N., Rodríguez-Pérez J.M., Barquera R., Gallardo G.J., Sánchez-Arenas R., García-Peña M.d.e.l.C., Granados J. (2008). Genetic admixture and diversity estimations in the Mexican Mestizo population from Mexico City using 15 STR polymorphic markers. Forensic Sci. Int. Genet..

[66] Iyer S.S., Cheng G. (2012). Role of interleukin 10 transcriptional regulation in inflammation and autoimmune disease. Crit. Rev. Immunol..

[67] Abdalla M.A., Hamdan H.Z. (2025). *IL13* gene polymorphisms among Sudanese patients with bronchial asthma: A case-control study. Mol. Biol. Res. Commun..

[68] Grunwald C., Adamska-Patruno E., Wawrusiewicz-Kurylonek N., Czarnowska A., Snarska K., Dardzińska-Głębocka A., Kapica-Topczewska K., Mirończuk A., Bazylewicz M., Kochanowicz J. (2023). Multiple sclerosis susceptibility may be associated with the coding rs20541 (R130Q) IL-13 gene polymorphism in the Polish population. Sci. Rep..

[69] Xie Z., Ding X., Wang Y., Zhang M. (2023). The rs2275913 polymorphism of the interleukin-17A gene is associated with the risk of ovarian endometriosis. J. Obstet. Gynaecol..

[70] Espinoza J.L., Takami A., Nakata K., Onizuka M., Kawase T., Akiyama H., Miyamura K., Morishima Y., Fukuda T., Kodera Y. (2011). A genetic variant in the IL-17 promoter is functionally associated with acute graft-versus-host disease after unrelated bone marrow transplantation. PLoS ONE.

[71] Borilova Linhartova P., Kastovsky J., Lucanova S., Bartova J., Poskerova H., Vokurka J., Fassmann A., Kankova K., Izakovicova Holla L. (2016). Interleukin-17A Gene Variability in Patients with Type 1 Diabetes Mellitus and Chronic Periodontitis: Its Correlation with IL-17 Levels and the Occurrence of Periodontopathic Bacteria. Mediat. Inflamm..

[72] Nie K., Zhang Y., Gan R., Wang L., Zhao J., Huang Z., Tang H., Wang L. (2013). Polymorphisms in immune/inflammatory cytokine genes are related to Parkinson’s disease with cognitive impairment in the Han Chinese population. Neurosci. Lett..

[73] Liu T., Yang L., Lv X., Zuo C., Jia C., Yang Z., Fan C., Chen H. (2022). Cumulative evidence for associations between genetic variants in interleukin 17 family gene and risk of human diseases. Front. Immunol..

