# Supplementary material for: Genetic Variants in Cytokines IL-10 and IL-17A Are Associated with the Risk and Severity of Sporadic Parkinson’s Disease in Mexican Patients
Source: Life (Basel). 2025 Sep 20;15(9):1480. doi: 10.3390/life15091480 (PMC12471379; doi:10.3390/life15091480)
Supplement: Supplementary file 1 [file life-15-01480-s001.zip › life-3854298-supplementary.pdf]

Supplementary Table S1. Characteristics of the genetic variants included in the study

Variant[¥]	Location: nucleotide change	Amino acid change	Functional effect / Reported associations
IL-10			
rs1800896	chr1-206773552-T-C<br>promoter region-Intron 1: (NM_153758.5):c.-149+2474T>C	NA	This variant is also known as -1082T>C. It has been associated with colorectal and prostate cancer, allergy and asthma exacerbations, and rheumatoid arthritis, as well. Homozygous individuals for the -1082C variant experience elevated levels of IL-10. PARP-1 was shown to specifically bind to the −1082 T haplotype in reporter assays and inhibit IL-10 promoter activity, thus providing an insightful model that may explain variability of IL-10 expression in the population at large [66].
rs1800872	chr1-206773062-T-G<br>promoter region-Intron 1: (NM_153758.5):c.-149+1984T>G	NA	Both variants, rs1800896 and rs1800872 (a.k.a −592 T>G), have been strongly associated with various serum levels of IL-10 *in vivo* [66].
IL-13			
rs20541	chr5-132660272-A-G<br>Exon 4: (NM_002188.3):c.431A>G	p.(Gln144Arg)	This SNV is associated with IgE levels in asthma, allergy and chronic obstructive pulmonary disease. In addition, it has been related to brain glioma [67]. Their results regarding risk for myocardial infarction and diabetes are conflicting. This missense variant occurs in α-helix D of the cytokine which is a critical region for interactions between IL-13 and its receptors, and therefore, it may have an impact on IL-13-mediated signaling, and may lead to inhibitory effects on immune responses, as it has been associated with multiple sclerosis [68].

IL-17A			
rs2275913	chr6-52186235-G-A<br>promoter region, 140 bp before transcription start site (NM_002190.3)	NA	This SNV is associated with higher serum levels of cytokine IL17A [69]. Espinoza et al. (2011) found that the A allele exhibited a higher affinity for the transcription factor NFAT and had a higher transcriptional activity of IL-17A than the G allele [70]. Borilova Linhartova et al. (2016) demonstrated that rs2275913 AA+AG carriers had higher IL-17A levels in mononuclear cells than GG carriers in chronic periodontitis patients [71]. It has been reported to be related to Parkinson's disease with cognitive impairment in the Han Chinese population [72]. A meta-analysis informed that this variant might fall in several putative regulatory regions (e.g., Histone modification of H3K4me1 and H3K27ac in blood, and enhancer histone modification of H3K4me3 in blood) [73].
rs8193036	chr6-52185695-C-T<br>promoter region, 2Kb upstream variant	NA	According to a prediction analysis, the C allele in this variant produces a DNA binding site for GATA-3 transcription factor with possible consequences on IL-17A expression [36]. The two SNV located in the IL-17A promoter were found not to be a risk factor of sporadic PD in a Polish population [13].

[¥]NCBI database of genetic variations, dbSNP. NA, not applicable. SNV, single nucleotide variant.

Supplementary Table S2. Odds ratio and relative risk values of gene frequencies between untreated patients and controls in the clinical group.

Genetic characteristic			Untreated PD patients in the clinical group (n= 26 and 52 alleles)			Controls in the clinical group (n= 21 and 42 alleles)			Statistical values	
**Gene**	**Variant ID**	**Genotype / allele**	**n**	**Frequency**	**CI 95%**	**n**	**Frequency**	**CI 95%**	**P-value**	**OR ($CI_{95\%}$) / RR ($CI_{95\%}$) values**
IL-10	rs1800896	TT	17	0.65	0.44–0.83	8	0.38	0.18–0.62	0.08	NA
		TC	9	0.35	0.17–0.56	8	0.38	0.18–0.62	1.00	NA
		CC	0	0	0.00–0.15	5	0.24	0.08–0.47	**0.01**	NA
		T	43	0.83	0.70–0.92	24	0.57	0.41–0.72	**0.01**	0.28 (0.11–0.72) / 0.52 (0.29–0.91)
		C	9	0.17	0.08–0.30	18	0.43	0.28–0.59		
	rs1800872	TT	15	0.58	0.37–0.77	5	0.24	0.08–0.47	**0.04**	4.36 (1.23–15.54) / 1.84 (1.09–3.10)
		TG	4	0.15	0.04–0.35	10	0.48	0.26–0.70	**0.03**	0.20 (0.05–0.78) / 0.43 (0.18–1.02)
		GG	7	0.27	0.12–0.48	6	0.28	0.11–0.52	1.00	NA
		T	34	0.65	0.51–0.78	20	0.48	0.32–0.64	0.10	NA
		G	18	0.35	0.22–0.49	22	0.52	0.36–0.68		NA
	Diplotype	TT-TT	12	0.46	0.29–0.65	4	0.19	0.07–0.41	0.06	3.64 (0.96–13.84) / 1.66 (1.03–2.68)
		TT-TG	2	0.08	0.01–0.25	4	0.19	0.07–0.41	0.39	NA

Genetic characteristic			Untreated PD patients in the clinical group (n= 26 and 52 alleles)			Controls in the clinical group (n= 21 and 42 alleles)			Statistical values	
Gene	Variant ID	Genotype / allele	n	Frequency	CI 95%	n	Frequency	CI 95%	P-value	OR ($CI_{95\%}$) / RR ($CI_{95\%}$) values
		TT-GG	3	0.12	0.03–0.29	0	0	0.00–0.18	0.24	NA
		TC-TT	3	0.12	0.03–0.29	1	0.04	0.00–0.24	0.45	NA
		TC-TG	2	0.08	0.01-0.25	6	0.29	0.14–0.50	0.12	NA
		TC-GG	4	0.15	0.06–0.34	1	0.05	0.00–0.24	0.36	NA
		CC-GG	0	0.00	0.00–0.15	5	0.24	0.10–0.45	0.01	NA
	Haplotype	H1	31	0.59	0.46–0.72	19	0.45	0.31–0.60	0.21	NA
		H2	11	0.21	0.12–0.34	11	0.26	0.15–0.41	0.63	NA
		H3	5	0.10	0.04–0.21	7	0.17	0.08–0.31	0.36	NA
		H4	6	0.12	0.05–0.23	5	0.12	0.05–0.25	1.00	NA
IL-13	rs20541	AA	13	0.50	0.30–0.70	8	0.38	0.18–0.62	0.56	NA
		AG	11	0.42	0.23–0.63	8	0.38	0.18–0.62	1.00	NA
		GG	2	0.08	0.01–0.25	5	0.24	0.08–0.47	0.22	NA
		A	37	0.71	0.57–0.83	24	0.57	0.41–0.72	0.19	NA
		G	15	0.29	0.17–0.43	18	0.43	0.28–0.59		NA
	rs2275913	GG	17	0.65	0.44–0.83	13	0.62	0.38–0.82	1.00	NA
		GA	8	0.31	0.14–0.52	6	0.28	0.11–0.52	1.00	NA
		AA	1	0.04	0.00–0.20	2	0.10	0.01–0.30	0.58	NA

Genetic characteristic			Untreated PD patients in the clinical group (n= 26 and 52 alleles)			Controls in the clinical group (n= 21 and 42 alleles)			Statistical values	
Gene	Variant ID	Genotype / allele	n	Frequency	CI 95%	n	Frequency	CI 95%	P-value	OR ($CI_{95\%}$) / RR ($CI_{95\%}$) values
IL-17A		G	42	0.81	0.67–0.90	32	0.76	0.61–0.88	0.62	NA
		A	10	0.19	0.10–0.33	10	0.24	0.12–0.39		
	rs8193036	CC	3	0.12	0.02–0.30	2	0.1	0.01–0.30	1.00	NA
		CT	9	0.35	0.17–0.56	5	0.24	0.08–0.47	0.53	NA
		TT	14	0.54	0.33–0.73	14	0.67	0.43–0.85	0.60	NA
		C	15	0.29	0.17–0.43	9	0.21	0.10–0.37	0.48	NA
		T	37	0.71	0.57–0.83	33	0.79	0.63–0.90		NA

Variant ID, from dbSNP database. These identifiers are assigned to unique variations within a reference genome. PD, Parkinson disease. n, number. CI, confidence interval. OR, odds ratio. RR, relative risk. Untreated patients (n= 26) and controls (n= 21) in the clinical group. NA, not applicable. Haplotype, H for *IL-10*_rs1800872/rs1800896. H1=TT, H2=TG, H3=CT, H4=CG. Significant p-values are shown in bold.

Supplementary Table S3. Association between genetic variants of cytokines (genotype and haplotype) and IL plasma levels, NLI and SII in patients and controls of the clinical group.

Gene (genetic variant)	Association	Mean 1±SD	Mean 2±SD	P value
IL-10 (rs1800896)	TT vs TC genotype with NLI in the patients of the clinical group	2.26±0.96	1.56±0.20	0.01
IL-10 (rs1800872)	TT vs TG genotype with SII in the patients of the clinical group	491.62±216.8	337.34±68.99	0.05
IL-10 (rs1800872)	TT+TG vs GG genotype with IL-10 in the controls of the clinical group	3.72±3.2	2.06±0.68	0.04

IL-10 haplotype	Reference diplotype TT-TT patients vs controls with SII	500.32±231.23	273.89±20.34	0.006
IL-10 haplotype	Reference diplotype TT-TT vs diplotype TC-TG with SII in the controls of the clinical group	273.89±20.34	576.28±110.71	0.0003
IL-10 haplotype	Reference diplotype TT-TT vs diplotype TC-GG with NLI in the patients of the clinical group	2.22±1.04	1.52±0.12	0.03
IL-13 (rs20541)	AA vs AG+GG genotype with IL-13 plasma levels	5.13±5.16	0	0.029
IL-17 (rs8193036)	CC + CT vs TT genotype with NLI in the patients of the clinical group	2.51±1.04	1.68±0.46	0.02
